# Endoscopic Ultrasound-Guided Gallbladder Drainage for Malignant Biliary Obstruction: A Systematic Review

**DOI:** 10.3390/cancers15112988

**Published:** 2023-05-30

**Authors:** Padraic McDonagh, Bidour Awadelkarim, John S. Leeds, Manu K. Nayar, Kofi W. Oppong

**Affiliations:** 1 HPB Medicine, Freeman Hospital, Newcastle Upon Tyne Hospitals NHS Foundation Trust, Newcastle Upon Tyne NE7 7DN, UK; padraic.mcdonagh@nhs.net (P.M.); bidour.awadelkarim@nhs.net (B.A.); j.leeds@nhs.net (J.S.L.); manu.nayar@nhs.net (M.K.N.); 2 Population Health Sciences Institute, Newcastle University, Newcastle Upon Tyne NE1 7RU, UK; 3 Translational and Clinical Research Institute, Newcastle University, Newcastle Upon Tyne NE1 7RU, UK

**Keywords:** endoscopic ultrasound-guided gallbladder drainage, malignant biliary obstruction

## Abstract

**Simple Summary:**

Endoscopic ultrasound-guided gallbladder drainage is emerging as an important technique for patients with malignant biliary obstruction where ERCP has failed. This meta-analysis is conducted to evaluate the efficacy and safety of this procedure. A comprehensive search of key databases was conducted, and all relevant studies were interrogated. A total of 7 studies were included in the final analysis with a total of 136 patients. The pooled clinical success rate of the procedure was 85%. Adverse events occurred in 13% of cases. No severe adverse events were reported. This meta-analysis supports the use of endoscopic ultrasound-guided drainage in malignant biliary obstruction when standard treatment has failed.

**Abstract:**

Background: Endoscopic ultrasound-guided gallbladder drainage (EUS-GBD) is a rescue technique for patients with malignant biliary obstruction who fail conventional treatment with ERCP or EUS-guided biliary drainage. The technique has been successfully employed in the management of acute cholecystitis in patients not fit for surgery. However, the evidence for its use in malignant obstruction is less robust. This review article aims to evaluate the data available at present to better understand the safety and efficacy of EUS-guided gallbladder drainage. Methods: A detailed literature review was conducted and several databases were searched for any studies relating to EUS-GBD in malignant biliary obstruction. Pooled rates with 95% confidence intervals were calculated for clinical success and adverse events. Results: Our search identified 298 studies related to EUS-GBD. The final analysis included 7 studies with 136 patients. The pooled rate of clinical success (95% CI) was 85% (78–90%, I^2^: 0%). The pooled rate of adverse events (95% CI) was 13% (7–19%, I^2^: 0%). Adverse events included: peritonitis, bleeding, bile leakage, stent migration, and stent occlusion. No deaths directly related to the procedure were reported; however, in some of the studies, deaths occurred due to disease progression. Conclusion: This review supports the use of EUS-guided gallbladder drainage as a rescue option for patients who have failed conventional measures.

## 1. Introduction

The majority of biliary strictures have a malignant etiology. Curative resection is only feasible in a minority of patients upon diagnosis due to the presentation of inoperable diseases, such as locally advanced tumors with vascular invasion, or metastatic diseases.

The focus of therapy in the majority of cases, therefore, is palliation. Endoscopic ultrasound-guided gallbladder drainage (EUS-GBD) is emerging as a rescue technique for managing malignant biliary obstruction, where first-line interventions have failed. This review evaluates the efficacy and safety of this procedure. Achieving biliary drainage is a key therapeutic aim in the management of patients with a malignant obstruction. In the majority of cases, whether operable or not, a biliary drainage procedure is required, either as the definitive management of the obstruction or as a stepping stone to resectional surgery. Endoscopically placed self-expanding metal stents are the standard of care [1]. Malignant biliary obstruction most commonly occurs in the setting of pancreatic ductal adenocarcinomas or cholangiocarcinomas. However, less common causes include gallbladder adenocarcinomas, duodenal malignancies, lymphomas, or metastatic lymph nodes [2]. Biliary obstruction leads to jaundice, which is often accompanied by pruritus and other debilitating symptoms. Patients may develop sepsis secondary to cholangitis. Palliative bile duct decompression is important for managing these patient’s symptoms.

Biliary drainage relieves itching, reduces the risk of cholangitis, and improves liver function. Biliary drainage is a prerequisite for the safe administration of palliative chemotherapy. Endoscopic retrograde cholangio-pancreatography (ERCP) is the first-line approach for achieving biliary drainage, but may fail in 5–10% of cases, even in expert hands [3]. Transpapillary access via ERCP can be challenging for a myriad of reasons: altered anatomy (Roux-en-Y, Whipple procedure, Billroth II surgery), gastric outlet obstruction, duodenal stricture, and periampullary diverticulae [4].

Percutaneous transhepatic cholangiography (PTC) has historically been the first-choice alternative; however, this is associated with an increased duration of hospitalization. Endoscopic ultrasound-guided biliary drainage (EUS-BD) is increasingly used and has the advantage of being able to be performed immediately following a failed ERCP, thereby offering cost and time savings. Recent studies have shown EUS-BD to be safe and effective following failed ERCP [5,6]. A retrospective multi-center study comparing surgery following EUS-BD with surgery following ERCP reported better outcomes following EUS-BD. This included a shorter duration between endoscopy and surgical interventions, higher rates of surgical clinical success, and a shorter length of hospital stay after surgery [7]. A recent meta-analysis has shown that EUS-guided drainage and PTC are equally effective; however, EUS-guided drainage is associated with a significantly lower rate of adverse events [8]. PTC often results in significant pain following the procedure; in many cases, an external drain is required that is less desirable or practical for patients. Therefore, EUS-BD has emerged as an important salvage technique when ERCP fails and, in the future, may be considered as a first-line intervention. EUS-GBD has been shown to be effective and safe in the management of acute cholecystitis in patients not fit for surgery [9]. In patients with a patent cystic duct, gallbladder drainage offers a potential alternative route to achieving biliary drainage. There is an increasing body of evidence to support its use in the management of refractory malignant biliary obstruction. EUS-GB drainage can also play a role when biliary drainage has been successfully established by ERCP or PTC stent insertion; however, the stent occludes the cystic duct orifice causing severe cholecystitis. In these cases, EUS-GB drainage can provide much needed relief from the symptoms of cholecystitis in a patient cohort with significant co-morbidities.

EUS-GBD is increasingly recognized as another endoscopic option that avoids the need for PTC drain insertion or surgery. Surgical drainage techniques, such as biliary enteric bypass, have very rarely been required in recent years, given the development of less invasive techniques. Biliary enteric bypass is associated with significant morbidity and mortality [10]. Importantly, surgical treatment is also associated with longer inpatient stays and increased costs, when compared with endoscopic management [11].

There are many nuances involved in the techniques involved in EUS-GBD. Endoscopic gallbladder drainage was first described in 2007; a diagnostic puncture of the gallbladder was performed, a wire was inserted, and the tract was dilated to facilitate drainage using pigtail stents [12]. With improvements in endoscopic ultrasound, the procedure is conducted, at present, under direct ultrasound guidance. In most cases, lumen-apposing metal stents (LAMS) are used [13]. The advent of LAMS has revolutionized the management of transmural pancreatic fluid collection. LAMS have a saddle shape and large lumen to facilitate drainage [14]. Several stent shapes and sizes exist, some with an electrocautery capped delivery system. LAMS are attractive for their relative ease of use and help to reduce procedure time [15]. The ESGE recommends the use of electrocautery-enhanced LAMS or dedicated SEMS for GB drainage [16]. Increasingly, LAMS have been used for an expanding range of interventions: EUS-guided gastrojejunostomy, EUS-guided hepaticogastrostomy, EUS-BD, and EUS-GBD. The route of access may be transduodenal or transgastric, depending on the gallbladder size, location, and availability of a blood vessel-free plane. Recent ESGE guidance recommends performing transduodenal EUS-GBD rather than transgastric, as this may reduce the risk of stent dysfunction (weak recommendation, low-quality evidence) [16]. A recent American Gastroenterological Association (AGA) clinical practice update advocated the placement of a double-pigtail stent across the LAMS to maintain stent patency, prevent migration, and contralateral wall injury [17,18]. There are also different methods of stent deployment: free-hand or wire-guided. Increasingly, LAMS are employed to achieve drainage using a freehand technique that avoids the need for fluoroscopy and saves time. As with any new technique, continued research is essential to evaluate the outcomes and identify the optimal method of achieving gallbladder drainage. The absolute contraindication to EUS-GBD is an established or suspected gallbladder perforation; when performing EUS-GBD it is preferrable to have a distended gallbladder to aid stent insertion. Other contraindications include the presence of large-volume ascites, biliary peritonitis, untreated coagulopathy, and a lack of a vessel-free plane for stent insertion. Anatomical variability can, in some instances, result in a gallbladder that does not approximate adequately close to the stomach or duodenum to allow drain insertion. EUS-GBD-specific adverse events include stent migration, gallbladder perforation, bleeding, and bile leak.

**Figure 1:**
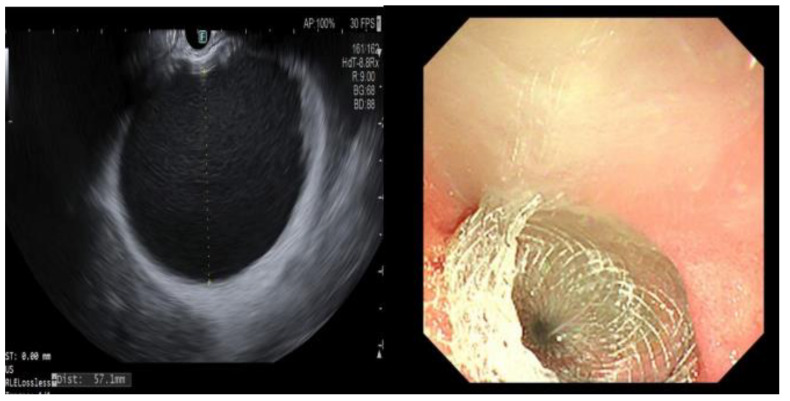
A 64-year-old man with metastatic cholangiocarcinoma. Primary tumor at the hilum; metastatic peritoneal deposits causing recurrent obstructive cholecystitis. Multiple admissions with abdominal pain and sepsis. EUS performed, gallbladder identified (**Left**), avascular plane located, and Hot Axios stent (10 × 15 mm) inserted (**Right**) with immediate drainage of purulent bile.

In addition to biliary drainage, EUS-GBD is also an option in the setting of post-stenting cystic duct occlusion or where tumor invasion/metastatic disease causes cystic duct blockage. This results in cystic duct dilatation, cholecystitis, and, in some cases, gallbladder rupture or perforation. EUS-GBD provides an option to safely control symptoms by providing gallbladder drainage in patients who are unfit for surgery. The procedure has been used for this indication in our institution with good clinical outcomes [19]. Where biliary drainage is the therapeutic aim, cystic duct patency is essential, whereas this is less important where gallbladder decompression is the goal (Figure 1).

EUS-GBD is used successfully in the management of patients with acute cholecystitis, who are not candidates for surgery. It has been used for the off-label indication for a number of years with excellent clinical outcomes and relatively few adverse events in a patient cohort with significant co-morbidities. In a randomized multi-center trial with 80 patients, those who underwent EUS-GBD had improved outcomes compared to percutaneous drainage [20]. The evidence for EUS-GBD in malignant biliary disease is less robust; however, in recent years, evidence has emerged supporting its use [21]. This review evaluates the evidence for and safety of EUS-GBD in patients with malignant biliary obstructions.

## 2. Materials and Methods

### 2.1. Literature Review

A comprehensive review of all the publications related to EUS-GBD for malignant biliary obstruction was conducted. Cochrane, PubMed, EMBASE, and Web of Science platforms were searched for any articles related to EUS-GBD until the 15 February 2023. The meta-analysis was conducted in accordance with the preferred reporting items for a systematic review and meta-analysis of diagnostic test accuracy studies (PRISMA) guidelines [22]. Only articles published in English were considered. One author (PMcD) conducted the screening of each database to obtain a list of the relevant articles. All articles related to EUS-GBD in the setting of malignant biliary obstruction were evaluated. The following search strategy was used, then repeated according to the specific syntax for each electronic database: (‘Endoscopic ultrasound’ OR ‘EUS’ OR ‘interventional Endoscopy’ AND ‘Gallbladder drainage’ OR ‘GBD’ OR ‘Cholecystogastrostomy’ OR ‘LAMS’ OR ‘Malignant biliary obstruction’ OR ‘Pancreatic cancer’ OR ‘Cholangiocarcinoma’).

### 2.2. Inclusion Criteria

All articles evaluating EUS-GBD as a salvage technique in patients with malignant biliary obstructions were included. The technical success of the procedure was defined as the successful deployment of the stent. Clinical success was defined as the resolution of jaundice or significant reduction in bilirubin (>50% reduction in bilirubin 2 weeks post-procedure). Adverse events included procedure-related bleeding, stent-related cholangitis, stent occlusion, stent migration, perforation, and bile leakage. Stent dysfunction was defined as the need for reintervention following stent insertion to improve symptoms or relieve obstruction. Articles were deemed irrelevant by title or, eventually, by abstract. Expert opinions, case reports, letters, and editorials were excluded. Where the research groups had multiple publications in the area, the most recent, largest, or most complete article was used. The full text of the remaining articles was evaluated.

The data extraction of included articles was performed by one author (PMcD). Key information recorded included: the study type, number of patients, baseline characteristics of the patient, level of biliary obstruction and etiology, stent type, route of access, complications, clinical success of the procedure, and duration of the procedure. A statistics software package (MedCalc version 20.218) using arcsine square-root transformation (Freeman–Tukey) was used to calculate the pooled rates with a 95% confidence interval for adverse events and clinical success. Heterogeneity was assessed using I^2^ statistic and Cochran’s Q. Egger’s test was used to assess for publication bias.

## 3. Results

### 3.1. Literature Search

**Figure 2:**
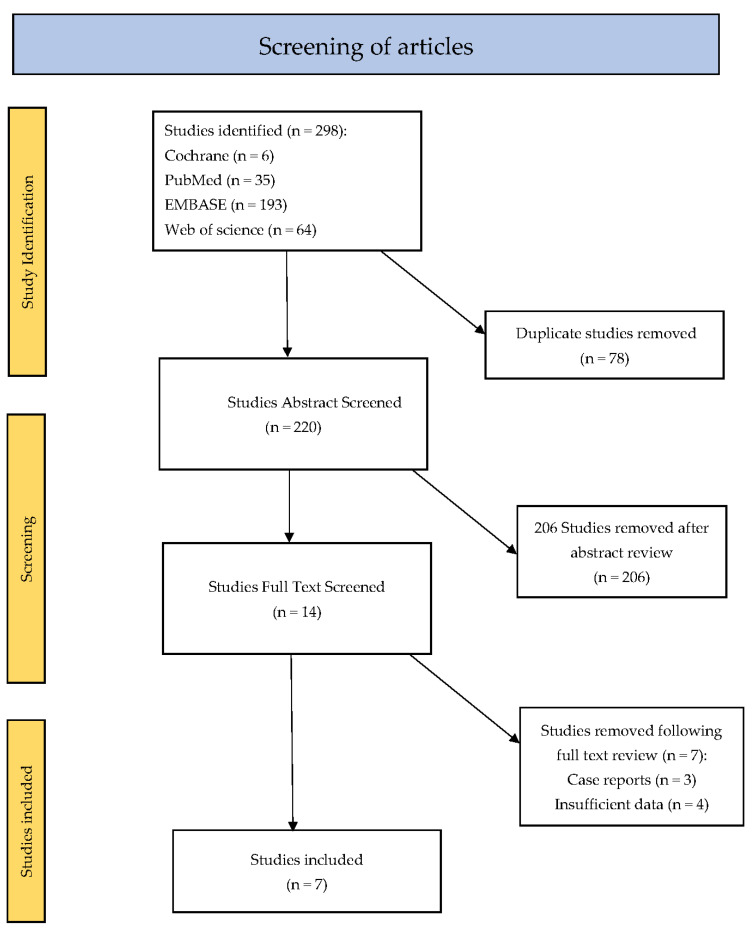
Flowchart of screening process.

Our search identified 298 studies related to EUS-GBD. Of these, seventy-eight duplicate studies were removed. A total of 220 study abstracts were interrogated, with 206 studies removed as they did not meet the inclusion criteria. Most of the studies removed involved research in the role of EUS GBD in acute cholecystitis. In some cases, studies evaluated both EUS-GBD and EUS-BD for malignant biliary obstruction; however, the data for the EUS-GBD cohort were not provided and, as a result, these studies were also excluded. This left fourteen studies where the full text was reviewed. Three of these were case reports and four had insufficient data and, as a result, were removed. This left seven studies included in the final analysis (Figure 2). The quality of the studies was deemed to be sufficient.

### 3.2. Clinical Details

A total of 136 patients were included across the seven studies. All studies included were retrospective in nature. The number of patients in each study ranged from 4 to 48 (Table 1). In all studies, EUS-GBD was attempted after failure at ERCP; in some cases, EUS-BD was also attempted prior to EUS-GBD. Only two of the studies discussed in detail the reasons for procedure failure [13,23]. ERCP was unsuccessful due to the suboptimal scope position, inability to advance a wire antegrade through the ampulla at EUS-BD, or due to abnormal duodenal anatomy [23]. In one paper from a center in Los Angeles, patients undergoing ERCP also consented for LAMS insertion (bile duct or gallbladder) for decompression, so that it could be achieved in one session [23]. None of the patients had undergone PTC or surgical biliary decompression. Technical success was achieved in all 136 cases. In the majority of cases, LAMS were used to achieve drainage, with the exception of a previous study from 2015, in which SEMS were deployed for gallbladder drainage [13]. In most cases, where electrocautery-enhanced LAMS were deployed, a freehand technique was used; however, this was left to the discretion of the endoscopist. An avascular plane was identified and the neck or body of the gallbladder was generally the ideal target [13]. The transduodenal approach was used in 45 (33%) of the patients, transgastric in 52 (38%) cases, and route of access was not recorded in the remainder of 39 (29%) cases [23,24,25]. In two of the studies, pigtail stents were used in some cases within the SEMS or LAMS to anchor the stent and prevent migration; however, this was not consistently reported upon [13,23]. Two studies highlighted the importance of evaluating cystic duct patency on imaging and EUS prior to stent insertion [13,23].

### 3.3. Meta-Analysis of Clinical Success and Adverse Events

**Figure 3:**
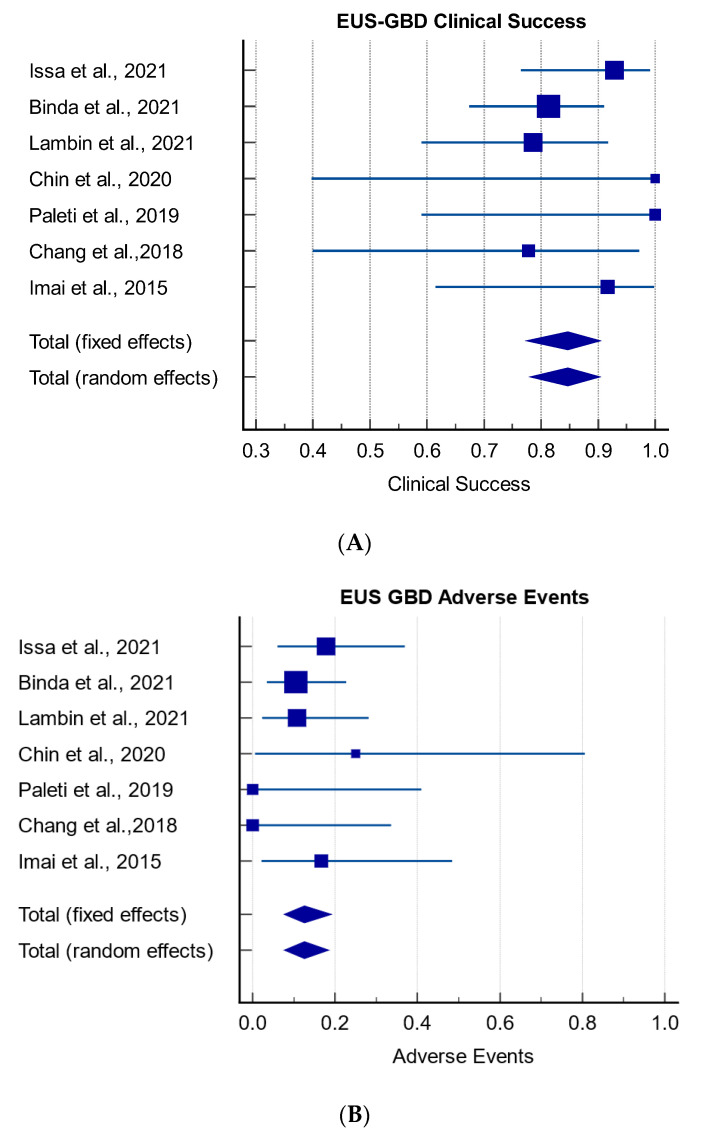
Rate of clinical success (**A**) and adverse events (**B**) in EUS-GBD for malignant biliary obstruction [13,21,23,24,25,26,27].

The pooled rate of clinical success (95% CI) was 85% (78–90%, I^2^: 0%). Clinical success was reported in all studies; in 5 of the 7, it was defined as a reduction in bilirubin of greater than 50% after two weeks [13,21,24,26,27] (Figure 3). In the remaining studies, it was described as a significant improvement in liver function tests [23,25]. The pooled rate of adverse events (95% CI) was 13% (7–19%, I^2^: 0%). Adverse events included: peritonitis, bleeding, bile leakage, stent migration, and stent occlusion. Adverse events were all managed conservatively. No deaths were reported to be directly related to the procedure; however, in some of the studies, deaths did occur due to disease progression [21].

## 4. Discussion

In some ways, EUS-GB for malignant biliary obstruction is a “back to the future” endoscopic recapitulation of a previously common surgical technique. Before the advent and widespread adoption of endoscopic biliary stenting via ERCP and the ready availability of PTC drainage as an option, surgical-loop cholecystenterostomy was a commonly performed palliative form of surgery [28]. Endoscopic stenting has historically been preferred to percutaneous stent for distal biliary obstruction following a randomized trial in 1987 that found that ERCP had a higher success rate and lower mortality [29]. A Cochrane meta-analysis in 2006 established that technical success and short-term efficacy were similar between ERCP and surgery for biliary obstruction due to pancreatic malignancy; however, morbidity and duration rates of hospitalization were higher for surgical bypass [30]. Therefore, ERCP, at present, is clearly established as the first-line palliative technique for distal biliary malignancy.

EUS-GB drainage re-establishes the option of gallbladder drainage, but endoscopically. The technique was initially described in the context of acute cholecystitis in high-risk surgical patients. In these cases, plastic pigtail and biliary self-expanding metal stents were used; these devices are not designed for this specific purpose and are therefore at risk of occlusion, leakage, and migration [31]. Additionally, their placement requires multiple steps and devices to puncture the gallbladder, dilate the tract, and finally place the stent, thereby increasing the complexity of the procedure and opportunities for failure and adverse events. The widespread availability of LAMS has led to an increased interest in the option of EUS-GB drainage for both benign and malignant indications. At present, EUS-GBD is a recognized and evidence-based option for high-surgical-risk patients with acute cholecystitis [20].

This meta-analysis demonstrated that EUS-guided gallbladder drainage for malignant biliary obstruction was technically and clinically successful in a high proportion of patients, either as a measure to facilitate neoadjuvant chemotherapy or as a definitive palliative treatment option. LAMS have been increasingly used for a number of off-label indications with a high degree of success. The ease of deployment and familiarity with the delivery system make it an excellent option when conventional methods have failed.

In this meta-analysis, 100% technical success was observed in stent delivery. Most of the procedures were performed in tertiary referral centers where a high volume of LAMS-insertion cases is conducted. In most cases, emphasis was placed on MDT discussions prior to stent insertion to ensure adequate patient selection. In particular, focus was given to evaluating for cystic duct patency, both on imaging and at the time of EUS [13,23]. Cystic duct patency is a key requirement in EUS-GBD where biliary drainage is the goal; imaging should be reviewed in all cases and the cystic duct takeoff interrogated closely to definitively exclude stones or obstructing mass. The route of access (transgastric or transduodenal) was usually based on the site that offered the best plane for stent insertion and was left to the discretion of the endoscopist. The majority of stents were inserted to maintain long-term biliary drainage; in some cases, a double-pigtail stent was used to prevent migration. Despite the heterogeneity in the techniques, equipment, and route used, the 100% technical success rate suggests that employing the approach the endoscopist is familiar with is reasonable. This study was not sufficiently powered to adequately compare the differences between the techniques used due to the low number of patients. There is a growing body of evidence to suggest that EUS-GBD is a safe technique; it has been deployed successfully in the management of patients with acute cholecystitis who are not candidates for surgery. In a meta-analysis of 1004 patients, a pooled technical success rate of 98% was observed and the pooled rate of adverse events was 14.8% [9].

The weakness of this study was the relatively low number of patients used. This is inevitable when the gold-standard treatment option (ERCP) is successful in over 95% of cases and where other salvage techniques are available. All the studies in the meta-analysis were observational and retrospective in nature, with a risk of increased confounding factors. There were also significant differences in the techniques employed to achieve gallbladder drainage. Different routes of access, stents, and anchoring devices were used. In some cases, a single endoscopist performed all operations and, in general, the procedures were conducted in tertiary referral centers. This leaves potential for bias; however, in real-world practices, given the technical complexities, these procedures are more likely be conducted in large tertiary centers. The strengths of this meta-analysis were the consistency in reporting of the clinical success rates and adverse events. Although there is a significant variation in patient demographics, it is a homogenous group with regard to the diagnosis of malignant biliary obstruction. The findings in this meta-analysis are also supported by the evidence that already exists for the use of the technique in acute cholecystitis [32]. The adverse event rate of 13% is also in line with what would be expected with a patient cohort presenting significant baseline morbidity.

Further research is required to clarify the best technique and equipment to use in EUS-GBD. Improvements in stent design and the quality of linear EUS scopes have dramatically improved the array of procedures the interventional endoscopist can provide; however, questions remain over which stents to use and what sizes. Should double-pigtail stents be inserted pre-emptively to prevent migration and impaction? With improvements in cancer therapy and patient survival, how will these stents be managed in the long term? Many challenges remain and continued innovation is required to adequately address these questions. EUS-GBD can be very technically demanding due to an unstable scope position or decompressed gallbladder. Novel solutions are required to overcome some of the technical difficulties of EUS-GBD. In many cases, the gallbladder does not approximate closely with the stomach or duodenum. This makes EUS-GBD impossible; however, the development of a retrievable puncture anchor traction system has been shown to aid gallbladder approximation in a porcine model. Successful gallbladder drainage was achieved in all animals in the treatment group following the use of the approximation device. Innovative and dynamic approaches similar to these are required to continue to push the boundaries of therapeutic EUS [33].

In this meta-analysis, the pooled clinical success rate was 85%. Clinical success was defined as the resolution of jaundice or a greater-than-50% reduction in bilirubin 2 weeks post-procedure. The clinical success achieved in this meta-analysis was similar to that achieved in EUS-guided biliary drainage [34]. This is impressive, given that this procedure was performed in a patient group who failed conventional management. ERCP has been the standard of care for decades and is a highly effective and successful technique. ERCP can achieve biliary drainage in over 95% of cases [35]. However, in certain situations, the likelihood of ERCP failure is increased (duodenal tumor invasion, altered anatomy, previous surgery) and, in these settings, EUS-GBD may provide an excellent salvage technique. The other options available to the patient include percutaneous drainage or surgical management. Although percutaneous drainage is an effective technique, it is associated with increased morbidity and even mortality. Percutaneous drainage of the gallbladder has been shown to have a morbidity outcome of up to 16% and adverse events in up to 40% of cases in a high-risk clinical group [36]. Adverse events included: bile leak, peritonitis, catheter leak, bleeding, and fistula formation. An external drain that is often a consequence of percutaneous drainage is also less desirable than internal drainage. Surgical drainage techniques are becoming increasingly rare, in part due to the success of endoscopic options. Biliary-enteric bypass is rarely performed due to higher rates of morbidity and mortality. In this meta-analysis, we achieved a clinical success rate of 85%, which supports the use of EUS-GBD as a salvage technique where ERCP and EUS-BD have failed.

In this study, the pooled adverse event rate was 13%. All adverse events were mild to moderate in severity and managed conservatively. These results are reassuring, given that the patient cohort in these studies had significant co-morbidities. There were no procedure-related deaths recorded; although, some deaths did occur due to disease progression. In a number of cases, reintervention was required for either further stenting or the insertion of pigtail stents to maintain drainage. In some cases, the data were lacking as patients became palliative and routine blood tests and reintervention became inappropriate.

## 5. Conclusions

Cancers that cause malignant biliary obstruction present a difficult clinical challenge. Achieving biliary drainage is essential to prevent jaundice, pruritus, coagulopathy, and sepsis. Although ERCP is successful in the majority of cases, rescue techniques are important where biliary decompression cannot be achieved through first-line methods. EUS-GBD has rapidly become established as the preferred option (when expertise is available) for high-risk surgical patients with acute cholecystitis and is emerging as a salvage technique in some cases of biliary obstruction. In this meta-analysis, EUS-GBD was observed to be a clinically successful and a safe option for patients with malignant biliary obstruction who failed conventional measures.

## Figures and Tables

**Table 1:** Main clinical details of studies included in meta-analysis.

	N	Study Type	No. of Males (%)	Stent Type	Route of Access	Adverse Events	Clinical Success
Issa et al., 2021 [21]	28	Retrospective	15 (54)	LAMS: 26 (92%), SEMS: 2 (7%)	Transduodenal: 15, transgastric: 13	5 (18%)	26 (93%)
Binda et al., 2021 [26]	48	Retrospective	23 (48)	LAMS: 48 (100%)	Transduodenal: 20, transgastric: 28	5 (10%)	39 (81%)
Lambin et al., 2021 [27]	28	Retrospective	N/A	LAMS: 28 (100%)	N/A	3 (11%)	22 (79%)
Chin et al., 2020 [24]	4	Retrospective	N/A	LAMS: 4 (100%)	N/A	1 (25%)	4 (100%)
Paleti et al., 2019 [25]	7	Retrospective	5 (71)	LAMS: 7 (100%)	N/A	0	7 (100%)
Chang et al., 2018 [23]	9	Retrospective	5 (56)	LAMS: 9 (100%)	Transduodenal: 5, transgastric 4	0	7 (77.8%)
Imai et al., 2015 [13]	12	Retrospective	8 (67)	SEMS: 12 (100%)	Transduodenal: 5, transgastric: 7	2 (17%)	(91.7%)

## Data Availability

Available on request from the author.
